# Gold Nanostars Embedded in PDMS Films: A Photothermal Material for Antibacterial Applications

**DOI:** 10.3390/nano11123252

**Published:** 2021-11-30

**Authors:** Gemma Toci, Francesca Olgiati, Piersandro Pallavicini, Yuri Antonio Diaz Fernandez, Lorenzo De Vita, Giacomo Dacarro, Pietro Grisoli, Angelo Taglietti

**Affiliations:** 1 Department of Chemistry, University of Pavia, Viale Taramelli 12, 27100 Pavia, Italy; gemma.toci01@universitadipavia.it (G.T.); francesca.olgiati01@universitadipavia.it (F.O.); psp@unipv.it (P.P.); yudhiistira@gmail.com (Y.A.D.F.); lorenzo.devita01@universitadipavia.it (L.D.V.); giacomo.dacarro@unipv.it (G.D.); 2 Department of Drug Sciences, University of Pavia, Viale Taramelli 14, 27100 Pavia, Italy; pietro.grisoli@unipv.it

**Keywords:** antibacterial materials, gold nanostars, photothermal effect, hyperthermia, PDMS

## Abstract

Bacteria infections and related biofilms growth on surfaces of medical devices are a serious threat to human health. Controlled hyperthermia caused by photothermal effects can be used to kill bacteria and counteract biofilms formation. Embedding of plasmonic nano-objects like gold nanostars (GNS), able to give an intense photothermal effect when irradiated in the NIR, can be a smart way to functionalize a transparent and biocompatible material like polydimethylsiloxane (PDMS). This process enables bacteria destruction on surfaces of PDMS-made medical surfaces, an action which, in principle, can also be exploited in subcutaneous devices. We prepared stable and reproducible thin PDMS films containing controllable quantities of GNS, enabling a temperature increase that can reach more than 40 degrees. The hyperthermia exerted by this hybrid material generates an effective thermal microbicidal effect, killing bacteria with a near infrared (NIR) laser source with irradiance values that are safe for skin.

## 1. Introduction

The development on surfaces of organized aggregates of microorganisms living within a self-produced matrix of extracellular polymeric substances, commonly referred as “biofilms,” has become a critical problem in many medical fields, as they represent a serious threat to the health of already fragile individuals [1]. As many studies report, the formation of the protective matrix enhances the resistance of the enclosed bacteria, making them much more resistant to conventional antibacterial and antimicrobial treatments [2]. This produced considerable interest from the scientific community in the development and production of biocompatible surfaces for medical use (prosthetic devices, catheters) suited for the prevention of the formation of biofilms or their eradication once biofilms have grown [3]. A recent, significant approach is the development of light-switchable materials to obtain surfaces in which hyperthermia can be produced by a remote light input by means of photothermal effects and then exploited to kill planktonic bacteria or eradicate already formed biofilms. In several examples, the photothermal properties are obtained using plasmonic noble metal objects, which are known to dissipate the intense absorption of light caused by localized surface plasmon resonance (LSPR) phenomena into thermal energy [4,5,6,7,8].

A material such as polydimethylsiloxane (PDMS) is indeed interesting for this approach because of its low cost, ease of fabrication, inertness against living tissue, and biocompatibility. These properties can be combined with antimicrobial ones to develop materials that can prevent the growth of bacteria and biofilm formation [9,10]; for example, it was shown how to load antimicrobial agents into PDMS, creating a time-sensitive device releasing the active agents as a function of time [11].

PDMS surfaces have also been studied as supports for the synthesis of thin films of metal nanoparticles and a monolayer of antibacterial agents for antibacterial purposes [12,13].

Keeping in mind the multitudes of uses that PDMS has been proved to have both in the pharmacological and technological field, the idea of embedding nanoparticles into the matrix could represent an innovative and useful method to combine the excellent transparency of materials obtained with this polymer with peculiar plasmonic properties of noble metal nano-objects. This topic was investigated by the work of Roper with a focus on heating applications such as plasmonic pervaporation [14,15]. PDMS foams functionalized with Au nanoparticles with applications to water remediation were also reported [16].

Non-spherical gold nanoparticles, such as gold nanorods (GNR) and gold nanostars (GNS), share with spherical gold nanoparticles (AuNP) already cited important optical features, with the ability to easily turn absorbed light into heat, when irradiated with a laser at a wavelength matching one of their LSPR bands. Moreover, anisotropic Au nano-objects possess the peculiarity that their LSPR bands can be placed easily in the desired region of the visible and near infrared (NIR) spectrum by tailoring the morphology of the object with synthetic manipulation. This feature is of particular importance for biomedical applications, as the NIR region between 750 and 1300 nm, which is called the “biological window,” is the one in which biological matter, such as living tissues, is more transparent to light penetration [17,18].

For this reason, a material containing GNS could be used in principle for antibacterial treatment of subcutaneous surfaces: the hyperthermia generated by the photothermal effects of GNS could be used to kill bacteria and destroy biofilms, and in proper conditions, this could also be performed on subcutaneous surfaces of prosthesis, catheters, and similar medical devices, provided that an intense LSPR absorption is correctly placed in the NIR biological window.

While several examples of the use of metal nanoparticles into a polymer framework for surface antibacterial applications have been reported [4,18,19,20,21], and while there are many advantages in using together PDMS and GNSs, to the best of our knowledge, the combination of the two to obtain on-demand hyperthermia to be exploited for antibacterial purposes has not yet been reported in the literature.

## 2. Materials and Methods

### 2.1. Materials

Gold(III) chloride trihydrate (~30 wt% in HCl 99.99%), sodium borohydride (98%), L-ascorbic acid (AA) (≥99%), silver nitrate (99.8%), sodium citrate (≥99%), hydrochloric acid (≥37%), nitric acid (1 N), TritonTM X-100, ethanol (≥99.7%), and MeO-PEG2000-SH were purchased from Aldrich (Burlington, MA, United States). Dowsil 184 Silicone elastomer kit was purchased from Dowcorning (Midland, MI, United States). All reagents were used as received. All the preparation are made with bi-distilled water.

Tryptone Soya Broth (TSB) and Tryptone Soya Agar (TSA) for bacteria culture were purchased from Oxoid, England. *S. aureus* ATCC 6538 and *E. coli* ATCC 10536 bacterial strains were used.

### 2.2. Instruments

UV-Vis-NIR absorption on PDMS samples were measured in air using a Varian Cary 50 UV/Vis spectrophotometer. The wavelength scan range was 300–1100 nm. The samples were placed in a special holder enabling transmission measurement of the same spot on the slide during all experimental stages.

Static contact angle determinations were made with a KSV CAM200 instrument with the water sessile drop method.

Transmission electron microscopy (TEM) images of the used GNS were taken on a Jeol JEM-1200 EX II instrument on 1:100 diluted GNSs solution, with a 10 μL sample dropped on nickel grids (300 mesh) coated with a Parlodion membrane.

Thickness of PDMS samples was checked with a Neotek Digital Thickness Gauge.

### 2.3. Methods

*Glassware Pre-Treatment.* All the glassware was usually pre-treated before use: a wash in *aqua regia* for 30 min, then washed and filled with bi-distilled water and ultrasonicated for 3 min before discarding water. The bi-distilled water/ultrasound treatment was repeated three times. Then the glassware was dried in an oven for 1 h at 140 °C. 

*Synthesis of Gold Nanostar (GNS).* GNS were prepared following the seed growth procedure previously described [17]. Briefly, seeds were prepared in a vial by adding 5.0 mL of TritonX-100 aqueous solution (0.2 M) and 5.0 mL of HAuCl_4_ aqueous solution (4.5 × 10^−4^ M). Then 600 µL of an ice-cooled solution of NaBH_4_ in water (0.01 M) were quickly added to the pale-yellow solution of AuCl_4_^-^ obtained in the previous step. The resulting brown-orange solution was gently hand-shaken for a couple of seconds; this solution was stored in an ice bath and needed to be used within 3 h. The growth solution was prepared starting from 50 mL of 0.2 M TritonX-100 solution in water and adding, in this precise order and under magnetic stirring, 2500 µL of AgNO_3_ in water (0.004 M), 50 mL of aqueous HAuCl_4_ (4.5 × 10^−4^ M), and 1600 µL of an aqueous L-ascorbic acid solution (0.0788 M). This process obtained a colorless solution just after a few seconds of gentle mixing. After this, 120 µL of seed solution was added, during which one could observe the suspension turning from pink to purple and blue, finally giving a gray-blue colloid. At this point, the mixing was stopped. The colloidal suspensions can be stored in the preparation flask in the dark and used for coating within a week.

*Coating of GNS with PEG-SH and EtOH Suspension.* Coating of GNS with MeO-PEG2000-SH was obtained by adding the PEG thiol to a flask of GNS colloidal suspension and stirring overnight. The thiol concentration was 2.0 × 10^−5^ M. Removal of Triton after coating was obtained with two cycles of centrifugation for 25 min at 13,000× *g* rpm. After the second centrifugation step, the pellets were redissolved in EtOH, centrifuged again, and finally redissolved in an EtOH volume, which allowed to obtain a 100-fold increase with respect to the initial colloid concentration.

*Preparation of PDMS Films*. Synthesis of blank PDMS samples was performed following the manufacturer’s instructions, mixing the elastomer and the cross-linker in a 10:1 proportion. Synthesis of PDMS with embedded GNS was performed by adding to the elastomer/cross-linker mixture the proper quantity of 100× colloidal suspension of coated GNS in EtOH calculated in order to obtain the desired concentration of GNS in the final samples. The mixture was stirred until the colloid was uniformly distributed, then the mixture was transferred to petri dishes of 3.5 cm diameter and placed in an oven at 45 °C for curing overnight. Quantities of the elastomer/cross-linker used were calculated in order to obtain final samples having 3.5 cm diameter and 0.2 mm thickness.

*Photothermal Measurements.* Thermograms were collected using a Thermocam FLIR E40 and using an L808P200 Thorlabs as a laser source (λ = 808 nm) using a spot of 1 cm of diameter, using a power ranging between 50 and 200 mW.

*Thermal Microbicidal Tests.* Antibacterial activity due to photothermal effects was investigated against *Staphylococcus aureus* ATCC 6538 (Gram+) and *Escherichia coli* ATCC 10356 (Gram−). The microorganisms were grown overnight in Tryptone Soya Broth at 37 °C. Washed cells were resuspended in Dulbecco’s PBS, and optical density (OD) was adjusted to 0.2 at 650 nm wavelength, corresponding approximately to 1 × 10^8^ colony-forming units (CFU)/mL. PDMS samples were cut in sections of about 10 mm diameter in order to be almost completely irradiated by laser. A volume of 0.01 mL of bacterial suspension was deposited on a coverglass slide, and the drop was covered with a PDMS sample. Contact and/or irradiation was performed for 30 min. After this time, the glass/PDMS section containing the bacterial suspensions was suspended in 1 mL of sterile water, gently shaken, and then water was suitably diluted in three different tubes: 1:100, 1:10,000, and 1:100,000. From each tube, 1 mL of suspension was taken and then cultured on Tryptone Soya Agar to count viable cells. The decimal-log reduction rate, i.e., the “thermal microbicidal effect,” ME was calculated with the following formula:

ME = log N_C_ − log N_E_

where N_C_ is the number of CFU/mL developed in contact (30′) with a control, not irradiated PDMS blank sample, and N_E_ is the number of CFU/mL counted after exposure (30′) to: (i) blank PDMS and irradiation (experiment “BL”), (ii) modified PDMS without irradiation (experiment “S”), and (iii) modified PDMS and irradiation (experiment “SL”).

## 3. Results

### 3.1. Preparation of PDMS Samples with Embedded GNS

**Figure 1:**
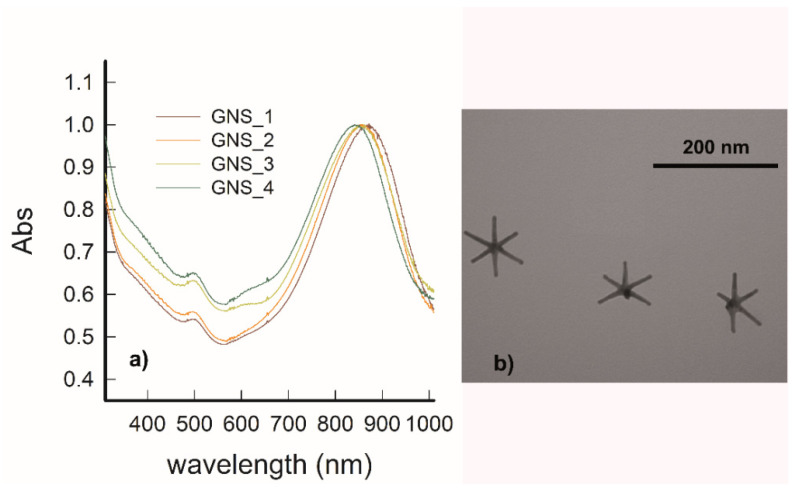
(**a**) LSPR spectra of four colloidal samples of GNS, normalized for intensities, showing the position of LSPR1 and the good reproducibility of the preparation. (**b**) TEM image of GNS from a representative preparation (scale bar, 200 nm).

GNS were prepared according to a synthetic pathway optimized in our laboratory [17], exploiting a seed growth process involving Triton X-100 surfactant as a shape-directing and protecting agent and AgNO_3_ in small amounts to further direct the anisotropic growth. The obtained GNS typically show 2–6 sharp branches surrounding a core. The synthetic parameters used allow obtaining GNS with 5–6 branches with a tip-to-tip maximum distance of 80–120 nm. These GNS have multiple LSPR absorption bands, dominated by two very intense peaks whose λ_max_ can be tuned, changing the synthetic conditions, between 750 and 1100 nm (for peak named LSPR1) and between 1200 and 1600 nm (LSPR2), respectively. In this study, we obtained the LSPR1 band placed between 840 and 870 nm, while the second was found at 1500–1600 nm (not shown in Figure 1). The weak peak around 510 nm is to be ascribed to the transversal oscillation of electrons, typical of all elongated gold nanoparticles, and to spherical by-products. As can be seen in Figure 1a, the synthetic conditions used allow for good reproducibility. A TEM image showing objects typically obtained in a representative preparation is reported in Figure 1b.

The weakly bound surfactant (Triton X-100) can be removed easily by means of substitution with a thiol-terminated capping agent; we opted for thiol-terminated polyethylene glycol (PEG-SH 2000) to ensure good solubility in water [22] but also miscibility in some organic solvents. Coating with PEG-SH 2000 resulted in a small (6–8 nm) blue shift of the plasmon peak LSPR1.

After the coating, the colloidal suspensions were purified by centrifugation and redissolved in EtOH. Dissolution in ethanol and the repeated centrifugation steps did not change the LSPR spectra, indicating good stability of coated GNS and no changes of the GNS morphology; however, it produced a small (ca 10 nm) redshift of LSPR1 due to the variation of the refractive index of the solvent, which changed from water (1.333) to EtOH (1.3611). After the third centrifugation/redissolution step, the pellet of coated GNS was redissolved in an EtOH volume to obtain a 100-fold concentration with respect to the original value.

At this point, we used the 100× concentrated suspension to prepare GNS embedded in PDMS samples. The setup of the preparation was organized in order to produce 3.5 cm diameter disks having a thickness of about 0.20 (0.02) mm. The 100× concentrated colloidal suspension was added in small aliquots to the mixtures of elastomer and cross-linker in order to obtain four different concentrations of embedded GNS: 0.9, 1.8, 2.7, and 3.6 × 10^−6^ mol of Au for g of elastomer. After prolonged stirring, the samples were placed at 45 °C overnight for the curing of the polymer. We observed that working with concentrations higher than 3.6 × 10^−6^ mol of Au/g of elastomer resulted in the failure of the formation of the solid PDMS sample after the curing step. This impossibility to obtain a solid sample when increasing the concentration of nano-objects too much was already reported for the case of spherical Au NP embedded in PDMS [20].

### 3.2. Characterization of PDMS Samples with Embedded GNS

**Figure 2:**
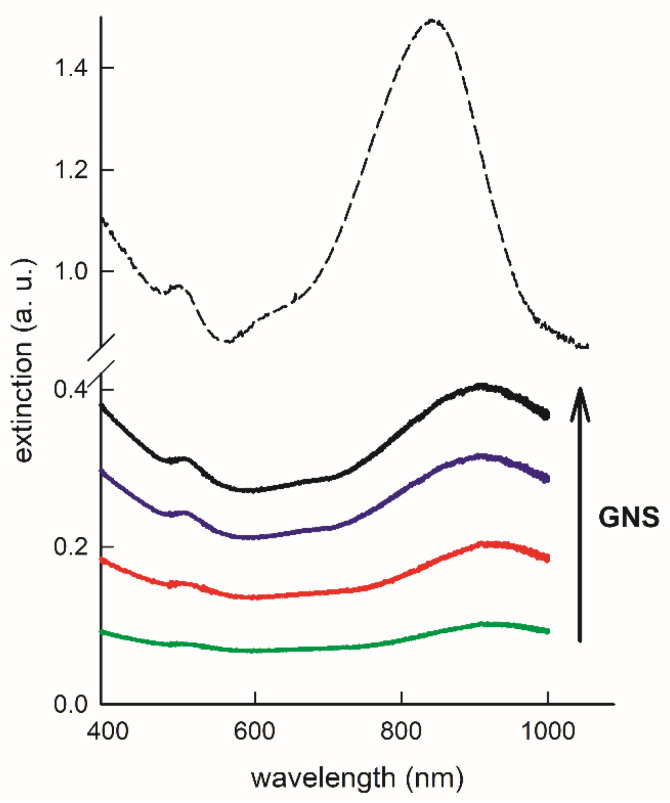
LSPR spectra of a colloidal suspension of coated GNS in EtOH (dashed line) and of four different PDMS samples with embedded coated GNS at increasing concentrations (solid lines, green: 0.9 × 10^−6^; red: 1.8 × 10^−6^; blue 2.7 × 10^−6^; black 3.6 × 10^−6^ mol Au/g of elastomer).

After the curing step, homogeneous disks were obtained, showing a deep dark blue color that increases with the increase in GNS concentration. Spectra obtained for the samples at different concentrations are reported in Figure 2 (solid lines), together with the spectrum of a colloidal 1× concentration GNS suspension in EtOH (dashed line).

Starting from the estimated value of 2.9 × 10^−16^ g of weight for a single GNS that we have already reported [22], we can estimate the presence of a number close to 6 × 10^11^ GNS for each gram of elastomer at the lower concentration used (0.9 × 10^−6^ mol of Au for g of elastomer). As expected, absorption of PDMS samples increases with GNS concentration. As can be clearly seen, we observed a further and consistent redshift independent from GNS concentration with respect to the case of GNS in EtOH solution. Again, this can be ascribed to the increase in the refractive index, which moves from the value of 1.3611 (EtOH) to 1.43 (PDMS). The overall effect of coating with PEGs and embedding in PDMS is that the LSPR1 wavelength moves into the range between 880 and 930 nm. Nonetheless, the LSPR features typical of GNS are conserved, indicating that no changes in the morphology of GNS have happened during the embedding and PDMS curing steps. In Figure S1, 10 spectra taken in 10 different points of a disk (concentration: 2.7 × 10^−6^ mol of Au for g of elastomer) are shown, indicating good homogeneity of the GNS dispersed in the polymer matrix of the disk. In Figure S2, for each one of the six prepared disks, the mean spectra obtained from the 10 different measures in different positions into a disk are reported, indicating good intersample reproducibility. Figure S3 reports the photograph of the same six disks.

We measured contact angles for all samples, including a reference PDMS disk prepared in the absence of embedded GNS. As can be seen in Table 1, no meaningful differences are present between the blank PDMS samples and PDMS samples containing increasing quantities of GNS.

In order to assess the stability of samples and to exclude the possible release of metals from the disks, five small pieces (about 0.20 g each, one for each concentration of embedded GNS) were immersed in 20 mL of bi-distilled water. After 24, 48, and 120 h of immersion, solutions were analyzed with ICP, indicating the absence (<5 ppb) of released silver and gold species in all the solutions.

The stability of samples was also demonstrated by taking spectra after 2 months of disks stored in air; for all samples investigated, no changes were registered in the features of extinction spectra and LSPR features, indicating the long-term stability of GNS in the PDMS matrix.

### 3.3. Evaluation of Photothermal Features

Having assessed the spectra of PDMS samples with increasing concentrations of embedded GNS, we moved to evaluate the photothermal behavior of the samples when irradiated in the NIR. We used an 808 nm laser, a source that we typically use for NIR irradiation for photothermal purposes. Although not perfectly matching the LSPR1 maxima of GNS inside the PDMS environment (which moved, from the original 840–870 nm range to values over 900 nm as a result of changes of the refractive index moving from water to PDMS as surrounding media), it ensures very good extinction efficiency. Thus, we considered it suitable to produce efficient hyperthermia.

**Figure 3:**
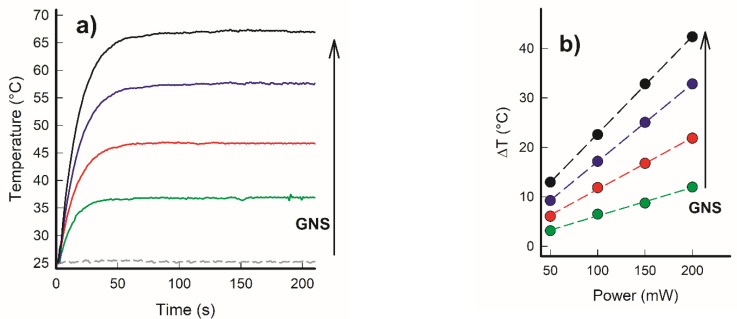
(**a**) Thermograms of five different PDMS samples with embedded coated GNS at increasing concentrations (0, 0.9, 1.8, 2.7, and 3.6 × 10^−6^ mol Au/g of elastomer) obtained by irradiation with an 808 nm laser at 200 mW of power (irradiance: 0.264 W/cm^2^). (**b**) ΔT vs. applied power for PDMS samples with embedded GNS at increasing concentrations, showing a linear dependence (green: 0.9 × 10^−6^; red: 1.8 × 10^−6^; blue 2.7 × 10^−6^; black 3.6 × 10^−6^ mol Au/g of elastomer).

One can appreciate the correctness of this choice in Figure 3a, in which representative thermograms obtained for four samples having different GNS concentrations (plus the blank PDMS sample) are reported. The behavior suggests a linear relationship between GNS concentration and the hyperthermia that can be obtained.

The temperature can be increased by more than 40 °C quite rapidly (in less than one minute) with the most concentrated sample. This was obtained using a power of 200 mW to irradiate a round spot of 1 cm diameter on portions of the disk having the dimensions. This corresponds to an irradiance of 0.264 W/cm^2^, which is below the limit considered safe for skin exposure [23]. All the samples were also irradiated using different laser powers, and in all cases, in the investigated range, we observed a linear relationship between the applied power and obtained temperature increase, as demonstrated by Figure 3b.

In addition, homogeneity of the samples was controlled, irradiating up to five different spots on a single sample and registering temperature increases, which was found to be extremely reproducible within a range of a few degrees, as can be seen in Figure S4.

**Figure 4:**
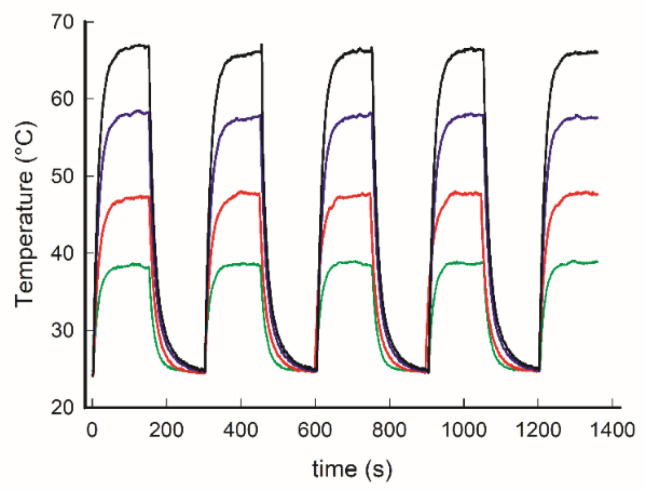
Thermograms for PDMS samples with increasing concentrations of embedded GNS (green: 0.9 × 10^−6^ mol Au/g of elastomer; red: 1.8 × 10^−6^; blue 2.7 × 10^−6^; black 3.6 × 10^−6^) on alternating 150 s irradiation ON with 150 s OFF.

All the four types of samples were subjected to four cycles of irradiation ON (150 s) and OFF (150 s), with subsequent rapid heating and rapid cooling, as shown in Figure 4, showing sturdy stability and reproducibility under the examined condition of samples for all the concentrations of GNS investigated.

### 3.4. Killing of Planktonic Bacteria

To evaluate the microbicidal effect of the described hybrid materials, we chose an intermediate value of GNS concentration, 2.7 × 10^−6^ mol Au/g of elastomer, which is already able to achieve hyperthermia of more than 30 °C. We used the same irradiation instrument used for photothermal characterization, an 808 nm laser, using 200 mW power, to irradiate an experimental setup consisting of a supporting thin glass that had a 10 μL drop of standardized solution of bacteria deposited upon. The drop was covered with a 1 cm diameter disk of the hybrid photothermal material. Irradiation was kept for 30 min.

We evaluated the microbicidal effect using the formula ME = log(N_C_) − log(N_E_), where N_C_ and N_E_ represent, respectively, the colony-forming units after contact in the control sample (PDMS without GNS and without irradiation) and in the proper experiment sample. We studied two different strains representative of the Gram-negative and Gram-positive bacteria *E. coli* and *S. aureus*, respectively.

We measured the ME for three different situations: for PDMS samples in the absence of GNS but under irradiation (sample blank + laser, BL), for PDMS with embedded GNS but in the absence of irradiation (sample star, S), and for PDMS with embedded GNS in the presence of irradiation (sample star + laser, SL). The results are given in Table 2.

As can be clearly noticed, irradiation in the absence of GNS or the presence of GNS but in the absence of irradiation does not produce any sensible antibacterial behavior for the tested strains, demonstrating that the ME is purely of photothermal origin. On the other hand, the effect of hyperthermia in the properly irradiated GNS-containing samples gave encouraging results for both strains. In particular, for *E. coli*, we obtained an ME higher than 4 (corresponding to the elimination of more than 99.99% of bacteria), while in the case of *S. aureus*, a lower but sensible effect was observed, indicating the elimination of about 90% of bacteria. The difference in the two values of ME can be related to the differences in the cell wall of the two strains: *E. coli* is a Gram-negative strain with a thinner and lipophilic cell wall, probably much more sensitive to heat and more prone to adhesion to the hydrophobic PDMS, resulting in a closer contact of bacteria cells to high-temperature surfaces. *S. aureus* instead has a more resistant and thicker cell wall, lesser adhesion to PDMS, and lower sensibility to hyperthermia, as already observed with similar functional surfaces [7].

## 4. Conclusions

In conclusion, we optimized an easy method to synthesize thin films of a PDMS-GNSs hybrid having LSPR absorption centered in the “biological window” that can be used for NIR irradiation to obtain hyperthermia, which can be easily modulated by changing the GNS concentration and laser power. We studied both the internal homogeneity and the reproducibility of the preparation. We characterized the photothermal effect and studied their effectiveness as microbicidal materials. Moreover, the obtained films are stable and safe, with no degradation of embedded nano-objects with use and time, and with no release of metal ions during the use.

The results obtained from the experiments conducted are promising, as we found a good photothermal-based microbicidal effect for both a Gram+ (*S. aureus*) and a Gram− (*E. coli*) bacterial strain. The microbicidal effect proved to be purely photothermal, and so it can be remotely activated on demand to destroy planktonic bacteria, which could lead to biofilm formation. Given that PDMS is a safe and biocompatible material widely used for prosthetic implants, we believe that the embedding of a photothermal material able to give such safe hyperthermia-based microbicidal action deserves special attention. The next step will be the assessment of its ability to eradicate and degrade formed biofilms.

## Figures and Tables

**Table 1:** Contact angle values for the PDMS samples containing increasing quantities of embedded GNS.

Au Concentration (×10^−6^ mol Au/g Elastomer)	Contact Angle (°) ^1^
0	110 (4)
0.9	115 (2)
1.8	114 (4)
2.7	117 (4)
3.6	108 (2)

^1^ mean of five measurements, s.d. in parenthesis.

**Table 2:** ME values obtained with a PDMS sample containing GNS concentration at 2.7 × 10^−6^ mol Au/g of elastomer. Samples named BL and SL were irradiated with an 808 nm laser with irradiance of 264 mW/cm^2^.

Experiment	ME
*S. aureus* BL	0.2 ± 0.1
*S. aureus* S	0.2 ± 0.1
*S. aureus* SL	1.0 ± 0.3
*E. coli* BL	0.1 ± 0.1
*E. coli* S	0.1 ± 0.1
*E. coli* SL	4.1 ± 0.5

## Supplementary Materials

The following are available online at https://www.mdpi.com/article/10.3390/nano11123252/s1, Figure S1: UV-Vis-NIR spectra taken in 10 different points of a PDMS sample (sample “b,” see Figure S3) with embedded GNS, Figure S2: UV-Vis-NIR spectra of six PDMS samples (samples a–f, see Figure S3) with embedded GNS, Figure S3: photograph of six different samples of PDMS with embedded GNS having concentration of 2.7 × 10^−6^ mol of Au for g of elastomer, Figure S4: thermograms obtained in five different spots of a PDMS sample.
