# Supplementary material for: Gold Nanostars Embedded in PDMS Films: A Photothermal Material for Antibacterial Applications
Source: Nanomaterials (Basel). 2021 Nov 30;11(12):3252. doi: 10.3390/nano11123252 (PMC8707359; doi:10.3390/nano11123252)
Supplement: Supplementary file 1 [file nanomaterials-11-03252-s001.zip › nanomaterials-1482765-supplementary.pdf]

nanomaterials

Supplementary Material

Gold Nanostars Embedded in PDMS Films: A Photothermal Material for Antibacterial Applications

Gemma Toci [1], Francesca Olgiati [1], Piersandro Pallavicini [1], Yuri Antonio Diaz Fernandez [1], Lorenzo De Vita [1], Giacomo Dacarro [1], Pietro Grisoli [2] and Angelo Taglietti [1,*]

[1] Department of Chemistry, University of Pavia, Viale Taramelli 12, 27100 Pavia, Italy; gemma.toci01@universitadipavia.it (G.T.); francesca.olgiati01@universitadipavia.it (F.O.); psp@unipv.it (P.P.); yudhiistira@gmail.com (Y.A.D.F.); lorenzo.devita01@universitadipavia.it (L.D.V.); giacomo.dacarro@unipv.it (G.D.)

[2] Department of Drug Sciences, University of Pavia, Viale Taramelli 14, 27100 Pavia, Italy; pietro.grisoli@unipv.it

* Correspondence: angelo.taglietti@unipv.it; Tel.: +39-382-987-342

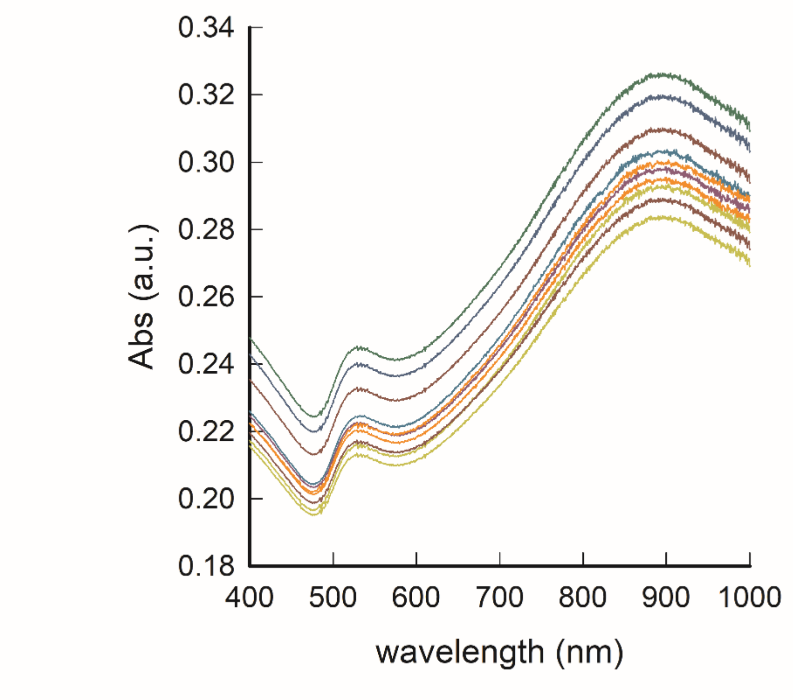

Figure S1. UV-Vis-NIR spectra taken in 10 different points of a PDMS sample (sample "b", see Figure S3) with embedded GNS (concentration: 2.7×10^{-6} mol of Au for g of elastomer).

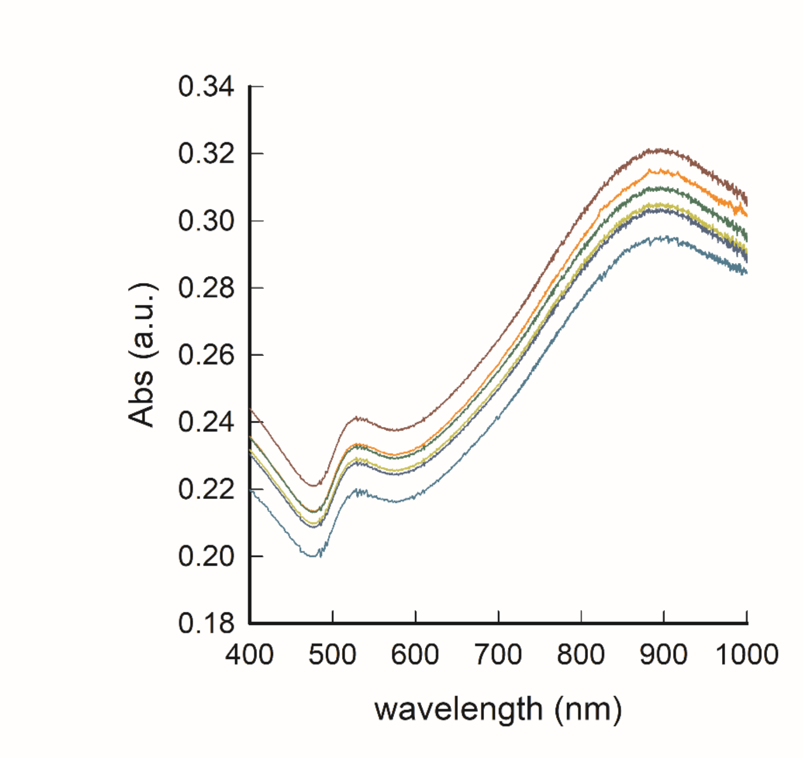

Figure S2. UV-Vis-NIR spectra of six PDMS samples (samples a-f, see Figure S3) with embedded GNS (concentration: 2.7×10^{-6} mol of Au for g of elastomer). Each spectrum is obtained as a mean spectrum from measurements in ten different points of each sample.

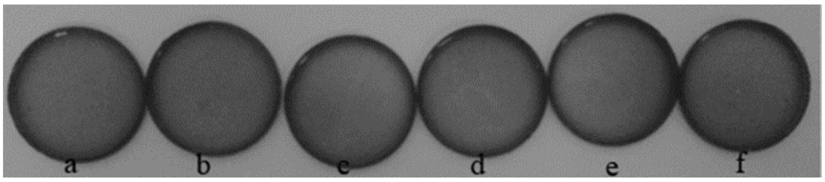

Figure S3. Photograph of six different samples of PDMS with embedded GNS having concentration of 2.7×10^{-6} mol of Au for g of elastomer.

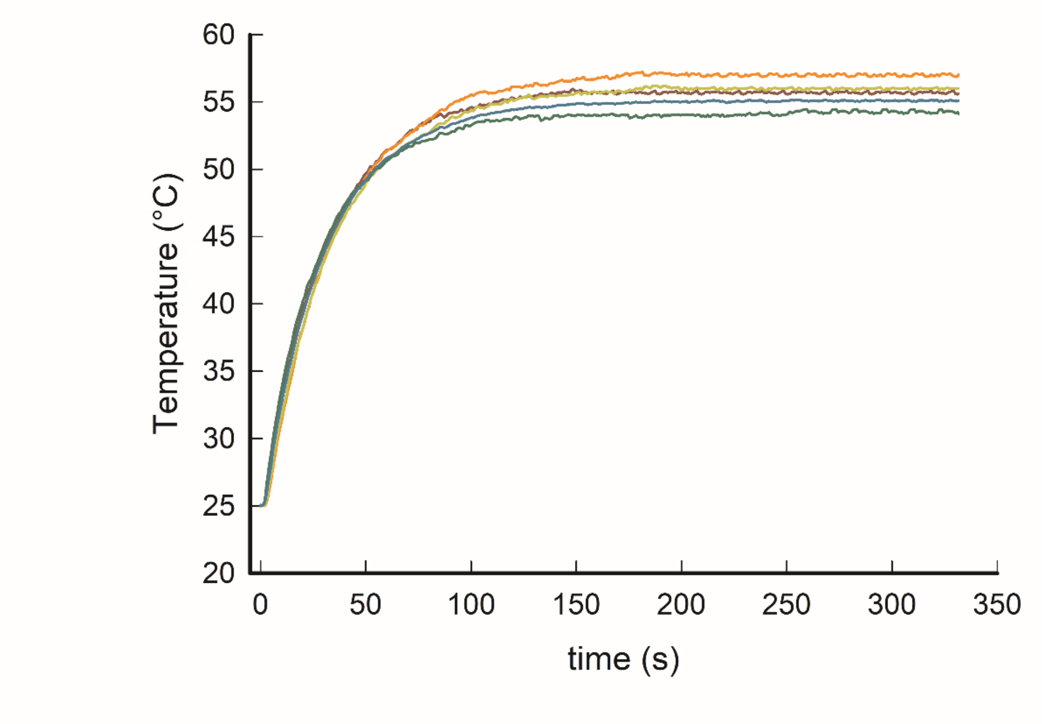

Figure S4. Thermograms obtained in five different spots of a PDMS sample (sample "a" in Figure S3) upon irradiation at 808 nm with irradiance 0.264 W/cm^2.
